# Editorial: Rising stars in developmental psychopathology and mental health 2024

**DOI:** 10.3389/frcha.2026.1937431

**Published:** 2026-08-25

**Authors:** Nicky Wright, Christine Puckering

**Affiliations:** 1 School of Psychology, Manchester Metropolitan University, Manchester, United Kingdom; 2 School of Health & Wellbeing, University of Glasgow, Glasgow, United Kingdom

**Keywords:** child and adolescent mental health, context, developmental mechanisms, developmental psychopathology, translation

Developmental psychopathology provides a framework for understanding how biological, psychological and social processes interact across development to shape mental health outcomes (1, 2). The studies within this collection illustrate this perspective by considering influences operating across multiple levels, from individual biological and behavioural processes, to parent-child relationships and family functioning, and broader contextual influences including culture and societal changes. The papers span infancy through adolescence, diverse international settings, and a wide range of methodological approaches, highlighting the breadth of contemporary research in developmental psychopathology and mental health. They demonstrate the creativity and innovation of emerging researchers working to advance understanding of developmental processes and inform prevention and intervention efforts.

Several contributions emphasise the importance of studying child development and mental health within the contexts in which they occur. Costantini et al. and Huntley et al. used innovative head-mounted camera technology to capture parent–child interactions and infant feeding experiences in naturalistic settings, providing new opportunities to examine developmental processes. Holla et al. highlighted the importance of understanding parenting experiences within the cultural context of urban India, while Yusuf et al. and Lees et al. examined child and family functioning during the unprecedented social disruption of the COVID-19 pandemic. Yusuf et al. identified distinct profiles of functioning among children with developmental delays and disabilities and demonstrated the importance of parenting self-efficacy in understanding variation in outcomes, while Lees et al. highlighted the protective role of family routines for both children and parents. Together these studies illustrate the value of considering context when understanding the complexities of child development and family life.

A second theme running through the collection is going beyond simple identification of risk factor and outcome and identifying mechanisms through which risk and resilience influence developmental outcomes. Several studies in the collection sought to understand the processes that may explain why mental health difficulties emerge and are transmitted across generations. Costantini et al. used innovative observational methods to identify early maternal and infant behaviours associated with maternal psychopathology assessed pre-birth. Using the same methodology, Huntley et al. tested whether differences in attention to food during parent-infant mealtimes may represent one mechanism involved in the intergenerational transmission of eating-disordered behaviours. Culpin et al. explored pathways linking paternal postnatal depression to child outcomes, examining the potential mediating role of fathers’ parenting behaviours. Similarly, Braithwaite et al. drew on hypotheses regarding HPA-axis functioning and sex-specific developmental adaptation to examine whether early environmental mismatch and infant tactile stimulation were associated with adolescent depressive symptoms differently for males and females. Mechanism was also central to the work of Tanner et al., whose evaluation of Mellow Babies explored the processes through which intervention-related change may occur. Collectively, these studies reflect a growing emphasis on understanding not only whether risk factors matter, but how they exert their effects on outcomes.

A third theme emerging from the collection is the importance of translating developmental science into interventions that are relevant, accessible and responsive to the needs of diverse populations. Hart et al. presented a protocol for evaluating an intervention targeting adolescent anxiety and depression in several African countries, addressing the need for evidence-based approaches that are both culturally appropriate and feasible to deliver within local contexts. Similarly, Holla et al.’s exploration of the needs of parents of toddlers in urban India highlights the importance of understanding the priorities and experiences of intended recipients when designing services and interventions. Two studies by Tanner et al. focused on the Mellow Babies programme, examining both participant experiences and processes of change. These studies demonstrate the value of moving beyond questions of efficacy alone to consider how interventions can be developed, adapted and implemented in ways that maximise engagement, acceptability and impact.

An overarching feature of the collection is the attempt to balance the identification of developmental mechanisms with the study of behaviour in ecologically valid settings. Across the papers, researchers sought not only to understand why mental health difficulties emerge, but to do so within the contexts in which children and families live their lives. This combination of mechanistic and contextually informed research is essential for ensuring that developmental science remains both theoretically informative and relevant to policy and practice.

The contributions in this Research Topic highlight the multifaceted nature of developmental psychopathology and mental health. They emphasise the importance of early experiences, family relationships, parental mental health and broader contextual influences across development, while also demonstrating the value of innovative methods and intervention-focused research. As a Rising Stars collection, the Research Topic showcases the breadth of expertise emerging within the field. The contributing authors address questions spanning infancy to adolescence and employ diverse methodological approaches across multiple cultural and geographical contexts. Collectively these studies demonstrate both the scientific quality and translational potential of research being led by the next generation of developmental psychopathology researchers. We hope this collection stimulates further research, collaboration and innovation within the field and supports ongoing efforts to improve mental health outcomes for children, young people and their families.
